# Ecological Factors Affecting Infection Risk and Population Genetic Diversity of a Novel Potyvirus in Its Native Wild Ecosystem

**DOI:** 10.3389/fpls.2017.01958

**Published:** 2017-11-14

**Authors:** Cristina Rodríguez-Nevado, Nuria Montes, Israel Pagán

**Affiliations:** ^1^Centro de Biotecnología y Genómica de Plantas – Universidad Politécnica de Madrid – Instituto Nacional de Investigación y Tecnología Agraria y Alimentaria, Madrid, Spain; ^2^Plant Physiology, Pharmaceutical and Health Sciences Department, Faculty of Pharmacy, CEU-San Pablo University, Madrid, Spain; ^3^Rheumatology Service, Hospital Universitario La Princesa, IIS-IP, Madrid, Spain

**Keywords:** virus infection risk, *Potyvirus*, population genetic diversity, plant–virus interactions, evergreen oak forests, virus ecology

## Abstract

Increasing evidence indicates that there is ample diversity of plant virus species in wild ecosystems. The vast majority of this diversity, however, remains uncharacterized. Moreover, in these ecosystems the factors affecting plant virus infection risk and population genetic diversity, two traits intrinsically linked to virus emergence, are largely unknown. Along 3 years, we have analyzed the prevalence and diversity of plant virus species from the genus *Potyvirus* in evergreen oak forests of the Iberian Peninsula, the main wild ecosystem in this geographic region and in the entire Mediterranean basin. During this period, we have also measured plant species diversity, host density, plant biomass, temperature, relative humidity, and rainfall. Results indicated that potyviruses were always present in evergreen oak forests, with a novel virus species explaining the largest fraction of potyvirus-infected plants. We determined the genomic sequence of this novel virus and we explored its host range in natural and greenhouse conditions. Natural host range was limited to the perennial plant mountain rue (*Ruta montana*), commonly found in evergreen oak forests of the Iberian Peninsula. In this host, the virus was highly prevalent and was therefore provisionally named mediterranean ruda virus (MeRV). Focusing in this natural host–virus interaction, we analyzed the ecological factors affecting MeRV infection risk and population genetic diversity in its native wild ecosystem. The main predictor of virus infection risk was the host density. MeRV prevalence was the major factor determining genetic diversity and selection pressures in the virus populations. This observation supports theoretical predictions assigning these two traits a key role in parasite epidemiology and evolution. Thus, our analyses contribute both to characterize viral diversity and to understand the ecological determinants of virus population dynamics in wild ecosystems.

## Introduction

Viruses are the most frequent causal agents of emerging infectious diseases in crops (Anderson et al., 2004; Woolhouse et al., 2005), and are responsible of yield losses that may have great economic and social impact (Oerke, 2006; Vurro et al., 2010). As a consequence, most of our knowledge on plant–virus interactions comes from the study of viruses that cause diseases in crops. However, viruses are widespread in wild ecosystems (Cooper and Jones, 2006; Roossinck, 2010; Prendeville et al., 2012), and there is increasing evidence indicating that they have co-evolved with their wild hosts long before they were domesticated (Lovisolo et al., 2003; Gibbs et al., 2008; Pagán and Holmes, 2010). The advent of next generation sequencing (NGS) has allowed exploration of the virome of wild plant communities (Roossinck, 2012; Stobbe and Roossinck, 2014), and these pioneering studies have shown that plant viruses in wild ecosystems are far more diverse than in agroecosystems. Thus, the detailed genomic and biological characterization of this viral diversity is central to fully understand plant–virus interactions (Pagán et al., 2016).

Plant viruses are not only highly diverse and widespread in wild ecosystems, they may also be important ecological agents. For instance, quantitative resistance of wild plants to viruses have been described (Gilbert, 2002; Pagán et al., 2010; Moreno-Pérez et al., 2014), suggesting that these may affect the host population composition. Also, viral infection can drastically reduce the number of individuals in the host populations by decreasing the competitive and reproductive abilities of infected plants (Anderson et al., 2004; Malmstrom et al., 2005; Vijayan et al., 2017). Although these studies show evidence that in wild host populations plant viruses may have great impact, little is known on their epidemiology and evolution and on the associated determinants (Pagán et al., 2016). The vast majority of the studies on this subject focused in plant viruses that are typical crop pathogens (Sacristán et al., 2004; Cooper and Jones, 2006; Webster et al., 2007; Pagán et al., 2010; Rodelo-Urrego et al., 2013; Tugume et al., 2016). However, crop viruses are not necessarily the most prevalent in wild ecosystems, and the impact of native plant viruses in the functioning of wild ecosystems could have been underestimated. For instance, wild ecosystems are abundant in long-lived perennial plants (Blanco et al., 2005). In contrast, agroecosystems are often dominated by annual plants – or managed as such –, and viruses adapted to wild perennials may over-compete crop viruses (Wren et al., 2006; Roossinck, 2010). In addition, human management of the host population has a great impact on plant virus populations (Pagán et al., 2012; Alexander et al., 2014; Rodelo-Urrego et al., 2015), so that plant viruses native from wild ecosystems may have different population dynamics than those typical in agroecosystems. Consequently, the factors driving the infection risk and population genetic diversity might be different than those identified for crop viruses (Fraile et al., 2017).

Analyzing the determinants of infection risk and population genetic diversity of native viruses in wild plant communities may be also relevant to understand their emergence. Emergent parasites are defined as those whose infection risk increases following its appearance in a new host population, or whose infection risk increases in an existing host population (Woolhouse, 2002). Consequently, understanding the determinants of parasite infection risk may contribute to understand the factors driving emergence (Keesing et al., 2006; Johnson et al., 2015). Because the rate of parasite emergence has accelerated in the last four decades (Anderson et al., 2004; Jones, 2009), understanding the determinants of emergence has become a long-standing goal of biological research. Changes in host ecology that increase infection risk have been proposed to be key determinants of emergence. Indeed, a recent theory known as the Dilution Effect links two such ecological factors – ecosystem species diversity and host density – to this process. This theory posits that reduced species diversity increases the density of the focal host species, facilitating parasite transmission and increasing infection risk, and eventually leading to emergence (Keesing et al., 2006; Ostfeld and Keesing, 2012). For plant viruses infecting wild hosts, experimental analyses of these predictions are scant, and do not always support the Dilution Effect (Malmstrom et al., 2005; Borer et al., 2010; Pagán et al., 2012). These contradictory results have been in part attributed to differences in virus traits such as host range and viral vectors. Consequently, the virus life history strategy – specialist (narrow host range) vs. generalist (wide host range) –, and ecological factors affecting vector populations (temperature, relative humidity, and rainfall) could also affect virus infection risk (Pagán et al., 2012). However, the role of these ecological factors in virus emergence has not been analyzed to date. Finally, it is important to note that ecological changes that affect infection risk may also result in genetic changes in the virus population (Grenfell et al., 2004; Archie et al., 2009; Pybus and Rambaut, 2009). Higher virus infection risk would lead to larger parasite population size, accelerating evolutionary rates (Scholle et al., 2013; Lanfear et al., 2014), which could ultimately result in higher population genetic diversity. Therefore, ecological factors that affect infection risk may also be important determinants of virus population genetic diversity. Again, this relationship has been seldom tested for plant–virus interactions in wild ecosystems (Rodelo-Urrego et al., 2015).

The focal wild ecosystem in this study is the evergreen Holm Oak (*Quercus ilex* L.) forest. This is the most extended wild ecosystem in the Mediterranean basin, covering approximately three million ha (Patón et al., 2009). In this geographical area, evergreen oak forests have a great ecological importance. They host annual and perennial plant communities that are typically associated with holm oak trees, and are also the refuge for the characteristic Mediterranean fauna (Lumaret et al., 2002). Evergreen oak forests have also great economic value, as they are appreciated as hunting reserves and holm oak acorns are used for animal feeding (Costa et al., 2011). Despite this ecological and economic relevance, the factors affecting virus infection risk and population genetic structure in evergreen oak forests remain largely unexplored. About 60% of Mediterranean evergreen oak forests are located in the Iberian Peninsula, where they represent the most frequent wild ecosystem (Ramírez and Díaz, 2008). Given that agricultural lands occupy 50% of the peninsula, evergreen oak forests are often adjacent to agroecosystems (Zamora et al., 2007), which favors plant virus dispersal between these two ecosystems (Alexander et al., 2014). Thus, analyzing virus diversity and population dynamics in this wild ecosystem may also be of interest to understand virus epidemics in crops.

We have focused in the species of the genus *Potyvirus* of plant viruses, which represent ∼30% of all known plant viruses. Potyviruses infect species from all major botanical families, and are transmitted by aphids (King et al., 2012). Most virus species in this genus are major crop pathogens (e.g., Walsh and Jenner, 2002; Quenouille et al., 2013), and some of them have been reported to infect wild plant species commonly found in evergreen oak forests (Malpica et al., 2006; Pagán et al., 2010). Together, these observations suggest that they may be important ecological agents in evergreen oak forests, and with potential to cause epidemics in crops. Potyviruses have tubular flexuous capsids, which encapsidate a monopartite single-stranded RNA genome ∼10,000 nucleotides. The genome is characterized by a single major open reading frame (ORF) encoding a large polyprotein that is processed into 10 functional proteins: the first protein (P1), helper component protease (HC-Pro), third protein (P3), 6K1, cylindrical inclusion protein (CI), 6K2, viral protein genome-linked (VPg), small nuclear inclusion protein (NIa), large nuclear inclusion protein (NIb), and coat protein (CP). Two additional proteins, P3N-PIPO and P3N-ALT, are originated through frameshifts in the P3 cistron (Chung et al., 2008; Hagiwara-Komoda et al., 2016), although they are not present in all members of the genus.

Here, we analyze the prevalence of species from the genus *Potyvirus* in evergreen oak forests of the Iberian Peninsula, as a measure of infection risk, based on surveys done between 2013 and 2016. This analysis identified a new potyvirus highly prevalent in its perennial host mountain rue (*Ruta montana* L.), commonly found in evergreen oak forests of the Iberian Peninsula. We provide information on the prevalence, genetic diversity and host range of this virus, provisionally named mediterranean ruda virus (MeRV). Using this host–virus interaction, we analyze the ecological factors affecting MeRV prevalence and population genetic diversity, and we explore the potential of MeRV for dispersing into crops by determining MeRV prevalence in cultivated fields located nearby evergreen oak forests where MeRV is present.

## Materials and Methods

### Field Sampling

**FIGURE 1:**
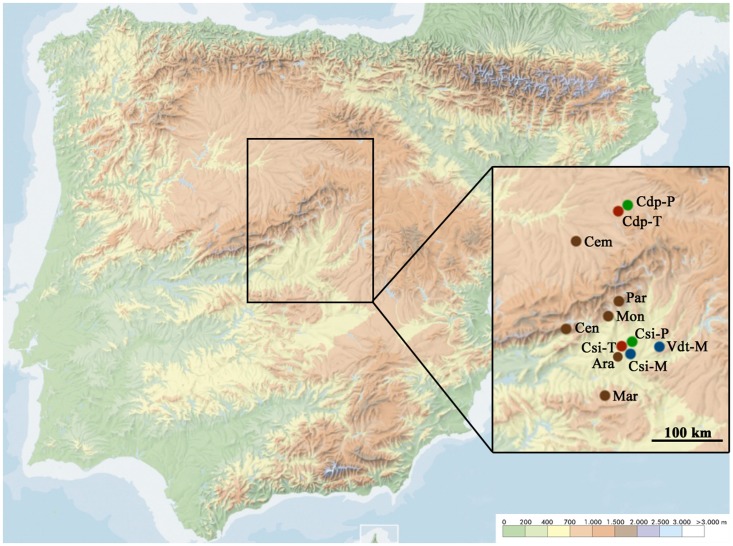
Geographic distribution of the evergreen oak forest and crop fields visited in this study. The locations of evergreen oak forests (brown), melon fields (blue), tomato fields (red) and pepper fields (green) are shown. Ara, Aranjuez; Cdp, Ciruelos de Pradales; Cem, Carbonero el Mayor; Cen, Cenicientos; Csi, Cortijo de San Isidro; Mar, Marjaliza; Mon, Montegancedo; Par, El Pardo; Vdt, Villamanrique de Tajo. Color scale in the bottom right of the image indicates the altitude gradient.

Six locations of evergreen oak forest located in the Iberian Peninsula were visited between the summer of 2013 and the spring of 2016. The locations were distributed among a transect of 200 km (north–south) in the center of the Iberian Peninsula (**Figure 1** and Supplementary Table S1). At each location, we defined a plot of 25 m × 25 m, which was divided in a grid of 1 m × 3 m. At each of these rectangles, leaves of one individual of the most abundant plant species (including herbaceous and non-herbaceous vegetation) were harvested. This sampling scheme allowed identifying the position of each sampled plant within the 25 m × 25 m plot, and therefore estimating plant density as the inverse of the mean Euclidean nearest neighbor distance (McGarigal et al., 2009). A total of 200 samples were collected at each location. Individuals representative of each collected sample were also harvested, inventoried in herbariums and their botanical family and species were determined. With this information, we calculated plant species richness (S) as the number of species at each location and visit, and plant species relative abundance (number of individuals of a given species/200). Samplings were performed in summer, autumn and spring to account for seasonal differences in plant species composition and phenological stage, and in aphid activity. A total of 10,800 samples were collected in these surveys. At each location and visit we also recorded maximum plant height and plant coverage in eight 1 m × 1 m squares within the plot. With these measures we calculated plant biovolume in the plot (m^3^) by averaging values in the eight squares. We also collected from nearby weather stations information on minimal, maximal and average temperature (°C) relative humidity (%), and on rainfalls (mm) in the months when each visit was done.

We also visited cultivated fields of melon (*Cucumis melo* L.), tomato (*Solanum lycopersicum* L.), and pepper (*Capsicum annuum* L.) located near to the sampled locations of evergreen oak forests (**Figure 1** and Supplementary Table S1). At least two fields from each plant species were visited in summer, spring, and autumn, and at each visit between 20 and 50 individuals were sampled. For this aim, three leaves of different branches from one out of every three plants were collected along a fixed itinerary.

### Potyvirus Detection and Identification

Infection by potyvirus species was detected in total RNA preparations from leaves using the cetyltrimethylammonium (CTAB) – polyvinyl pyrrolidone (PVP) method (Chang et al., 1993), which allowed efficient RNA extraction from all the collected plant species. The presence of virus species within the genus *Potyvirus* was analyzed by One-step SYBR Green-based real-time RT-PCR in the LightCycler^®^ 480 Real-Time PCR System (Roche Diagnostics, Mannheim, Germany). For each run, 10 ng of total RNA were added to the Brilliant III SYBR^®^ Green Ultra-Fast QRT-PCR Master Mix (Agilent Technologies, Santa Clara, CA, United States) following the manufacturer’s recommendations. Universal primers NIb2F and NIb3R (Zheng et al., 2010) for species of the genus *Potyvirus* were used to amplify a region of ∼350 nucleotides of the gene that encodes the NIb. The thermal profile consisted of a 5-min pre-incubation step at 65°C, 10-min RT step at 50°C and 5 s of Taq polymerase activation at 95°C, followed by 50 cycles of PCR at 95°C for 10 s (denaturation), 50°C for 20 s (annealing), and 72°C for 30 s (extension). RNA purified from turnip mosaic virus (TuMV), a well characterized potyvirus species, was always included as positive control. Amplification was confirmed in electrophoresis in 1.2% agarose gel stained with ethidium bromide in Tris-acetate-EDTA (TAE) buffer. PCR products of the expected length were purified using the StrataPrep DNA Gel Extraction Kit (Agilent Technologies) and sequenced. Sequences were assembled using MEGA 6 (Tamura et al., 2013). Nucleotide identity of the obtained sequences with the potyviral sequences available in GenBank was analyzed using BLAST^1^. Following the ICTV criteria (King et al., 2012), sequences with a nucleotide identity over 55% with any known species of the genus *Potyvirus* were considered as belonging to this genus, and sequences with nucleotide identity between 55 and 76% were considered as belonging to a non-previously described species of this genus (Adams et al., 2005). Total potyvirus prevalence was calculated as the percentage of infected individuals relative to the total number of analyzed plants. Prevalence for each virus species was calculated as the percentage of infected plants relative to the total number of plants collected from a given host species.

### Biological Characterization of MeRV

To determine the host range and the symptoms induced by MeRV infection, different plant species of the botanical families *Amaranthaceae*, *Asteraceae*, *Boraginaceae*, *Chenopodiaceae*, *Cistaceae*, *Cucurbitaceae*, *Fabaceae*, *Fagaceae*, *Poaceae*, *Rutaceae*, and *Solanaceae* were inoculated (**Table 1**). These species were chosen to include the most abundant species in the sampled evergreen oak forests, and the most common crops in the surrounding areas. For each plant species, four to seven plants (10–15 days old) were mechanically inoculated by applying purified virion RNA (100 ng/ul) in 0.1 M Na_2_HPO_4_ onto the first two completely expanded leaves dusted with carborundum. The inoculated plants were maintained in a greenhouse (20–25°C, and 16 h of light), and symptoms were weekly recorded over an 8-week period. Tissue from systemically infected leaves of symptomatic and asymptomatic plants was collected 20 days post-inoculation and tested for potyvirus infection by real-time RT-PCR using specific primers qCPFor (5′-GACTGACTATAGTTTAGCGCGC-3′) and qCPRev (5′-GCCTCTGATAGCTGCTGCTTTC-3′) that amplify a 111-bp region of the MeRV CP gene.

**Table 1:** Host range and symptoms of MeRV-ParP17.

Plant species	Systemic infection^1^	Systemic symptoms
*Amaranthaceae*		
*Gomphrena globosa*	0/6	–
*Asteraceae*		
*Lactuca sativa*	0/6	–
*Santolina rosmarinifolia^∗^*	0/6	–
*Taraxacum officinalis^∗^*	0/6	–
*Boraginaceae*		
*Anchusa azurea^∗^*	0/7	–
*Chenopodiaceae*		
*Chenopodium amaranticolor*	0/6	–
*Chenopodium quinoa*	0/5	–
*Cistaceae*		
*Cistus ladanifer^∗^*	0/6	–
*Cucurbitaceae*		–
*Cucumis melo*	0/5	–
*Cucumis sativus*	0/5	
*Fabaceae*		
*Anthyllis vulneraria^∗^*	0/5	–
*Lupinus angustifolius^∗^*	0/6	–
*Phaseolus vulgaris*	0/5	–
*Pisum sativum*	0/6	–
*Vicia villosa^∗^*	0/6	–
*Fagaceae*		
*Quercus ilex^∗^*	0/4	–
*Poaceae*		
*Bromus rubens^∗^*	0/6	–
*Rutaceae*		
*Ruta montana*	6/6	Asymptomatic
*Solanaceae*		
*Capsicum annuum (Dulce Italiano)*	4/6	Mild mosaic
*Nicotiana benthamiana*	5/5	Mild mosaic
*Nicotiana clevelandii*	0/5	–
*Nicotiana tabacum (Samsun)*	0/5	–
*Solanum lycopersicum*	6/6	Mild mosaic


*^1^Values are number of infected over inoculated plants.*

*Asterisks indicate plant species present in evergreen oak forests and coexisting with mountain rue (*Ruta montana)*.*

### Sequencing of the MeRV Genome

Total plant RNA of a single field-collected mountain rue (*Ruta montana* L.) from El Pardo that tested positive for MeRV infection (MeRV-ParP17) was treated with TURBO DNA-*free*^TM^ Dnase (Life Technologies, Carlsbad, CA, United States). After treatment, ribosomal RNA (rRNA) was removed using Ribo-Zero^TM^ Plant Leaf kit (Epicenter-Illumina, Madison, WI, United States). For genome sequencing, 3 μg of total RNA was used for library preparation and subjected to high-throughput NGS using the Illumina platform (HiSeq2000, 2 × 125 bp length, at the Centre for Genomic Regulation, Barcelona, Spain), generating 20 million paired-end reads. Adapters and low-quality sequences from NGS data were removed using Seqtk^2^. MeRV genome was assembled using a reference-guided read mapping to the phylogenetically nearest potyvirus genomes as implemented in Bowtie 2.0 (Langmead and Salzberg, 2012). BLAST was used to identify and remove possible chimeric reads contained in the alignment during the assembly. The consensus sequence of the MeRV genome was extracted using the *Fasta Alternate Reference Maker* tool in the Genome Analysis ToolKit (GATK) (McKenna et al., 2010). Importantly, no other viral sequences were detected in NGS data, in accordance with RT-PCR and virion purification analyses (see Results).

The NGS-derived MeRV genomic sequence was confirmed using the same mountain rue plant RNA preparation as template for RT-PCR, utilizing different sets of specific primers. Primer pairs were designed to produce 12 fragments in such a way that adjacent fragments overlapped by at least 100 nt, covering the potyvirus polyprotein (Supplementary Table S2). Expand^TM^ Reverse Transcriptase (Sigma–Aldrich, St. Louis, MO, United States) was used for retrotranscription and PCR Phusion^®^ High-Fidelity DNA Polymerase (New England BioLabs, Beverly, MA, United States) for PCR amplification. The 5′ and 3′ ends of the virus were obtained by rapid amplification of cDNA ends (RACE) using Ambion^TM^ FirstChoice^®^ RLM RACE kit (Life Technologies). All fragments were sequenced, and assembled with MEGA 6, revealing 100% nucleotide identity between overlapping fragments and with the NGS-derived nucleotide sequence. The same nucleotide sequence was also obtained using RNA from purified virions as template.

The ORF of the MeRV polyprotein was identified with ORF Finder^3^. PredictProtein (Rost et al., 2004) and the NCBI’s conserved domain database (CDD)-Search service (Marchler-Bauer et al., 2007) were used to identify the putative cleavage sites and the conserved sequence domains of the polyprotein. Molecular weight of the putative viral proteins was predicted from protein sequence by using the *Molecular Weight* tool^4^. The genomic sequence of MeRV-ParP17 was deposited in GenBank under accession number MF953305.

### Phylogenetic Analyses

Phylogenetic relationships between MeRV and the other members of the genus *Potyvirus* were analyzed using the nucleotide and amino acid sequences of the polyprotein. To study these relationships a collection of the reference sequences of the potyviruses compiled from GenBank were obtained (Supplementary Table S3). Nucleotide and amino acid sequences of the reference strain of each potyvirus were aligned with the corresponding sequences of MeRV-ParP17 using MUSCLE (Edgar, 2004). Alignments were used to construct phylogenetic trees utilizing Bayesian Markov Chain Monte Carlo (MCMC) methods as implemented in MrBayes v3.2.6 (Ronquist and Huelsenbeck, 2003). Alignments were run using the general time-reversible substitution model with invariant sites and a gamma distribution of among-site rate variation (GTR+I+Γ_4_) for nucleotides, and the Whelan and Goldman (WAG) substitution model for amino acids. All analyses were run until relevant parameters converged, with 25% of the MCMC chains discarded as burn-in. Maximum clade credibility (MCC) trees, with Bayesian posterior probability values providing a measure of the robustness of each node, were also summarized from the MrBayes tree samples.

Nucleotide identities (%) of the complete MeRV genome with those of the two phylogenetically closest virus species were compared using SimPlot v3.5.1 (Lole et al., 1999). Plots of nucleotide identity were obtained using the MeRV genome as the query sequence, and a sliding window of 200 nt that was moved across the alignment in steps of 20 nt.

### Genetic Diversity and Selection Pressures in the MeRV Population

The genetic diversity of the MeRV population was estimated based on the CP gene sequence of 69 MeRV isolates (Acc No. MF953306-MF953374). For this purpose, total RNA extracts of MeRV-infected plants were used to RT-PCR amplify the viral CP gene (837 nt), utilizing specific primers CPFor (5′-CCAAAGCTTGAACAAGAGAGAATTGTTTCG-3′) and CPRev (5′-ACACCAAGCATGKTRTGCATAT-3′), and PCR products were sequenced. Virus genetic diversity (π) as average pairwise nucleotide difference between sequences, and the mean number of non-synonymous (*d_N_*) and synonymous (*d_S_*) nucleotide substitutions per site, were estimated using the Kimura-2-parameter nucleotide substitution models implemented in MEGA 6, as this was selected as the best-fitted nucleotide substitution model in jModelTest v. 2.1.10 (Darriba et al., 2012). Selection pressures were estimated as the *d_N_*/*d_S_* ratio. Standard errors of each measure were based on 1,000 bootstrap replicates. Also, the number of MeRV haplotypes (H), and the haplotype diversity [H_d_ = (1 - Σ*x_i_*^2^)*n*/(*n* - 1), where *x*_i_ is the frequency of an haplotype and *n* is the sample size (Nei and Tajima, 1981)] were calculated using DnaSP v.5 (Librado and Rozas, 2009).

### Detection of Recombination in the MeRV Population

Potential recombination breakpoints in the CP and NIb genes were detected using either the MeRV sequences from 69 isolates or including sequences from these genes of all known potyviruses. Recombination was detected utilizing four different methods based on different assumptions (Posada, 2002) as implemented in the RDP4 package^5^: RDP, GENECONV, Bootscan, and Chimera, and employing the default parameters and a Bonferroni correction *P*-value cut-off of 0.05 (Martin et al., 2015). Only recombination signals detected by all methods were considered as positive to minimize false positives. With this criterion, no MeRV recombinants were detected. Analyses using the more relaxed criterion of considering as recombinants those detected by two or more methods to minimize false negatives yielded the same result.

### Statistical Analyses

Differences in host susceptibility and in MeRV prevalence between seasons in the same sampling year (summer, autumn, and spring), between sampling years at each season, and between geographic locations were compared using Chi-square tests. These statistical analyses were done using SPSS 21.0 (SPSS, Inc., Chicago, IL, United States). Mixed effect multiple regression tests were used to analyze the association between ecological factors and MeRV prevalence and population genetic diversity parameters (Burnham and Anderson, 2002). We considered the following factors as predictors of MeRV prevalence: host plant density and relative abundance, species richness, plant biomass, temperature, relative humidity and precipitations in the sampled locations (minimal, maximal, and average values), and season. The same ecological factors, with the addition of MeRV prevalence, were used as predictors of virus population diversity parameters. A set of models that included a global model containing all ecological factors as fixed predictors (except season that was considered as covariate), and nested models that contained all possible combinations of these predictors, were fitted for each response variable using general linear mixed models (R-library: ASreml-R 3). Models were constructed using a simultaneous autoregressive variance–covariance matrix to account for time-dependency and covariation between predictor variables. Global and nested models were ranked according to Akaike’s Information Criteria (AIC), and the model with the lowest AIC score was selected as the best-ranked model. The relative importance of the predictors included in each model was calculated by variance components (R-library: ASreml-R 3).

## Results

### Potyvirus Prevalence in Evergreen Oak Forests and Isolation of a New Virus Species

Infection by potyvirus was detected in every season during the monitored period, and prevalence was calculated as the percentage of infected individuals relative to the total number of analyzed plants. Average potyvirus prevalence in evergreen oak forests of the Iberian Peninsula ranged between 1.06 and 3.43%, depending on the season and sampling cycle. In the analyzed sampling cycles, potyvirus prevalence was higher in summer than in spring and autumn (χ^2^ ≥ 5.03, *P* ≤ 0.025), prevalence being similar in the later two seasons (χ^2^ ≤ 1.58, *P* ≥ 0.208).

Identification of the potyviruses present in the 171 infected plants indicated the presence of five virus species: *Zucchini yellow fleck virus* (ZYFV) (34/171), which was detected in six host plant species with a prevalence ranging between 4.76 and 61.54%; *Clover yellow vein virus* (ClYVV) (10/171), which was detected in two plant species with prevalence of 2.86–13.33%; and *Endive necrotic mosaic virus* (ENMV) and *Turnip mosaic virus* (TuMV) (2/171 each), which were found infecting individuals of a single plant species each, with prevalence of 3.51% and 4.65%, respectively. Interestingly, the remaining identified potyvirus accounted for the largest fraction of infected plants (123/171, 71.93%). This virus species was only detected in mountain rue (*Ruta montana*), a perennial plant that showed the highest potyvirus prevalence (41%) among the host plant species identified in evergreen oak forests. None of the other detected potyvirus was found in mountain rue plants. The comparison of the NIb of this rue-infecting virus with those of all known potyviruses revealed a nucleotide sequence identity of 75% with the closest species. This suggested that this was a novel species of genus *Potyvirus* that we provisionally named MeRV. For further characterizing the virus, tissue of one mountain rue plant sampled in El Pardo and infected with MeRV was selected. This plant tested negative for other species of the genus *Potyvirus* and for cucumber mosaic virus (CMV) by RT-PCR. More importantly, analyses of the NGS sequence data obtained from total RNA extracts of this field-infected mountain rue plant did not detect any other viral sequence. Tissue of the infected field plant was grinded in 0.1 M phosphate buffer pH 7, 0.02% sodium diethyldi-thiocarbamate (DIECA), and used to inoculate *Nicotiana benthamiana* plants. After 20 days, *N. benthamiana* plants developed a systemic mosaic. MeRV virions were purified from these plants according to the protocol described by Sánchez et al. (2013). Agarose gel electrophoresis of the virion preparation showed a single band, indicating that all virions in the preparation had the same size. Indeed, agarose gel electrophoresis of nucleic acid extracts from these virions showed a single band of ssRNA of about 9,000–10,000 nt (not shown). The same NIb nucleotide sequence amplified from the field-infected mountain rue plant was obtained from virion preparations using universal potyvirus primers. Together, these results indicated that a single virus species was transferred from mountain rue to *N. benthamiana* plants, and that this species belonged to the genus *Potyvirus*, discarding the possibility of mixed viral infection. This isolate was named MeRV-ParP17, and its virions were used for further characterization.

### Analysis of the Complete Nucleotide Sequence of MeRV

**FIGURE 2:**
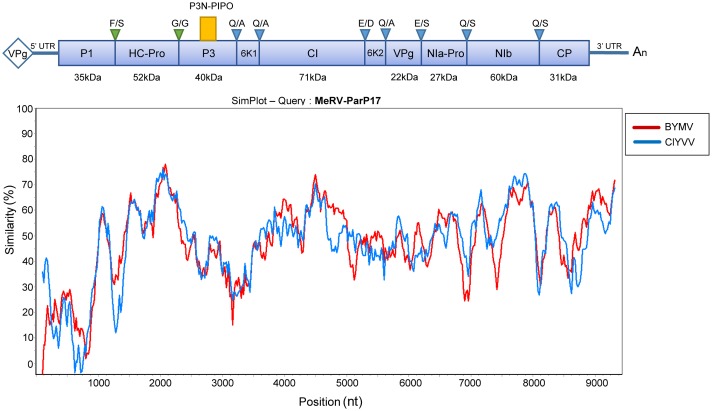
Schematic representation of the MeRV genomic organization and nucleotide identity with the phylogenetically closer virus species. The position of each gene is indicated in relation to the sequence of the isolate MeRV-ParP17 (GenBank acc. no. MF953305). The blue box represents the polyprotein ORF. The alternate P3N-PIPO ORF is depicted by the small yellow box above the main reading frame. The corresponding predicted molecular weights in kilodalton (KDa) are indicated below each polyprotein cistron. Autocatalytic cleave sites of the HC-Pro and P1 gene products are indicated by green triangles, and NIa-Pro cleavage sites are marked by blue triangles. Conserved amino acids at each cleavage site according to Adams et al. (2005) are shown above each triangle. The panel below plots the nucleotide similarity (%) between the MeRV-ParP17 genome and those of the phylogenetically nearest potyviruses: *Bean yellow mosaic virus* (BYMV) and *Clover yellow vein virus* (ClYVV). Each point plotted is the per cent identity within a sliding window of 200 nt wide centered on the position plotted, with a step size between points of 20 nt.

The MeRV genomic sequence was obtained by NGS sequencing and confirmed by RT-PCR. The virus full-length genome sequence consists of 9,560 nt excluding the 3′ terminal poly(A) tail. As for all members of the genus *Potyvirus*, the MeRV-ParP17 genome has a single ORF encoding a polyprotein of 3,077 amino acids with an estimated molecular weight of 350 kDa. This ORF encodes all typical proteins, consensus cleavage sites and conserved catalytic domains present in the potyviruses (**Figure 2**). The P1 protein, a serine protease, contains the strictly conserved catalytic triad His_208_-(X_8_)-Asp_217_-(X_30_)-Ser_248_ with the conserved GHSG_246-249_ motif around the active serine site. In the HC-Pro, the protease active site residues are located at Cys_344_, contained in the conserved sequence GYCH_342-345_, and at His_417_. Also, a conserved cysteine-rich region was identified in the N-terminal region of HC-Pro stretching from Cys_27_ to Cys_58_. HC-Pro motifs involved in aphid transmission (RITC_52-55_ and PTK_310-312_), genome replication (IGN_152-154_) and systemic movement (CCC_292-294_) (Urcuqui-Inchima et al., 2001) are also conserved in MeRV-ParP17. The P3 cistron contains the P3N-PIPO ORF. The 5′ end of this ORF starts with the highly conserved motif among potyviruses G_2_A_6_ and it has a length of 70 codons, terminating in a UAG stop codon at nucleotides 672–674 of the P3 cistron. The cylindrical inclusion (CI) protein cistron includes the conserved RNA helicase motifs located between residues 85 and 359, and the NIa contains the conserved His_46_, Asp_81_, Cys_151_, His_167_ cysteine protease catalytic tetrad. Conserved sequence motifs in RNA-dependent-RNA polymerases of positive strand RNA viruses were also identified in the MeRV-ParP17 NIb protein. Finally, the CP cistron contains a NAG motif, essential for aphid transmission in some potyviruses (Wylie et al., 2002), and located at residues 10–12 in the N-terminal region of the CP. The Arg_170_ and Asp_214_ residues, involved in virion assembly and cell-to-cell movement (Dolja et al., 1994, 1995), are located in the conserved core region of the MeRV-ParP17 CP. The virus 5′ untranslated region (UTR) is 149 nt long, an average length for potyviruses (<200 nt). The 3′ UTR is 180 nt long excluding the poly(A)-tail and is rich in AU segments, which is a common feature among potyviruses.

**FIGURE 3:**
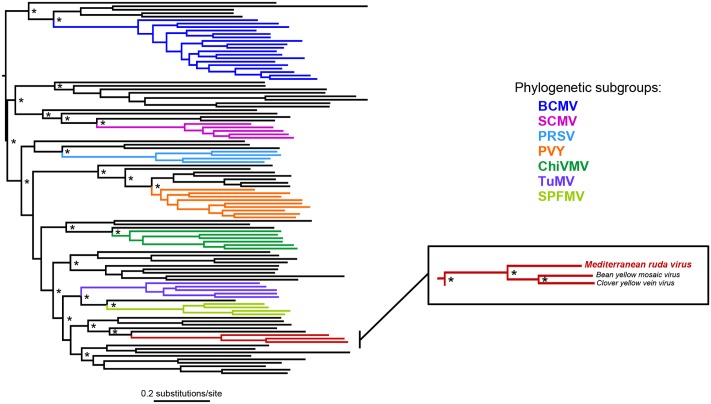
Bayesian phylogeny based on the coding nucleotide sequence of potyviruses. Asterisks indicate nodes with posterior probabilities of ≥0.90. The tree is mid-point rooted. Branch lengths are drawn to a scale of nucleotide substitutions per site. Phylogenetic subgroups of potyviruses defined by Shukla et al. (1994) are used as reference, and indicated in colored branches. BCMV, bean common mosaic virus; SCMV, sugarcane mosaic virus; PRSV, papaya ringspot virus; PVY, potato virus Y; ChiVMV, chilli veinal mottle virus; TuMV, turnip mosaic virus; SPFMV, sweet potato feathery mottle virus. Expansion of the phylogeny section contains the group of virus sequences in which MeRV is clustered.

The complete nucleotide sequence of the MeRV-ParP17 polyprotein was aligned with those of available fully sequenced potyviruses (*n* = 107). The percentage of nucleotide identity of the MeRV-ParP17 genome with that of the other potyviruses ranged from 50 to 66%, showing highest identity with bean yellow mosaic virus (BYMV) (65%) and clover yellow vein virus (ClYVV) (66%). Accordingly, Bayesian phylogenetic trees revealed that MeRV-ParP17 clustered together with BYMV and ClYVV (**Figure 3**). Following the approach described by Mbanzibwa et al. (2011) SimPlot analyses were performed to compare the percentage of nucleotide identity between MeRV-ParP17 and BYMV/ClYVV across the whole genome (**Figure 2**). When each cistron was analyzed individually, the MeRV-ParP17 P1 and P3 cistrons showed the lowest nucleotide identity with the same cistrons of BYMV and ClYVV (**Figure 2**). The remaining cistrons showed a sequence identity with the corresponding ones of BYMV and ClYVV ranging on average between 57 and 69%. Equivalent results were obtained using the amino acid sequences (not shown).

### MeRV Host Range

The MeRV-ParP17 host range was determined by inoculating purified virions of MeRV-ParP17 into 10 wild species belonging to seven botanical families, which represent the most abundant species in evergreen oak forests, and 13 plant species of six botanical families, including *Cucurbitaceae* and *Solanaceae* species that are commonly cultivated or found in the area surrounding the surveyed evergreen oak forests. The results are shown in **Table 1**. None of the 10 wild species from evergreen oak forests were hosts of MeRV, except its original reservoir mountain rue (6/6 plants infected), in which virus infection was asymptomatic. Only three *Solanaceae* species were infected by MeRV-ParP17: *N. benthamiana* (5/5), *C. annuum* (4/6), and *S. lycopersicum* (6/6), with infection in these three species causing mild mosaic symptoms. There was no variation in susceptibility across hosts (χ^2^ = 0.81, *P* = 0.668).

### Analysis and Ecological Determinants of MeRV Infection Risk in Evergreen Oak Forests

We used MeRV prevalence as a measure of virus infection risk. MeRV prevalence in mountain rue plants of Iberian evergreen oak forests did not significantly varied between geographic locations (χ^2^ ≤ 2.74, *P* ≥ 0.098), and this factor was not considered in subsequent analyses. On the other hand, MeRV prevalence varied across seasons between 18% in spring 2015 and 74% in summer 2014 (**Table 2**). Average MeRV prevalence did not vary between sampling cycles. However, during the first and second cycle the prevalence was higher in summer than in spring and autumn (χ^2^ ≥ 11.43, *P* < 1 × 10^-5^), whereas in the third sampling cycle the highest prevalence occurred in autumn (χ^2^ ≥ 4.79, *P* ≤ 0.029) (**Table 2**). Given that the MeRV host range included two crop species, tomato and pepper, commonly cultivated near the sampled evergreen oak forests, we analyzed MeRV prevalence in these crops during the same time span. We also included melon fields in our surveys, as, together with pepper and tomato, this was the major cultivated plant in the area. MeRV was never detected in melon tomato and pepper plants.

**Table 2:** Average values of MeRV prevalence and of ecological factors measured in evergreen oak forests locations included in the study.

Season	Prevalence (%)	Relative abundance	Plant density	Biovolume (m^3^)	*S*	T_max_	T_min_	T_mean_	RH_max_	RH_min_	RH_mean_	Rainfalls
Sampling cycle 1												
Summer 2013	46.15	0.075	2.940	0.013	24	34.47	8.80	22.40	86	18	39	95
Autumn 2013	33.33	0.150	2.363	0.047	15	24.90	-1.45	12.70	90	23	53	216
Spring 2014	40.00	0.085	2.360	0.046	26	28.37	2.87	14.50	89	19	46	230
Sampling cycle 2												
Summer 2014	73.91	0.120	2.542	0.009	25	36.30	11.33	23.70	86	18	37	42
Autumn 2014	31.25	0.240	2.435	0.045	18	29.10	5.93	16.90	89	26	57	419
Spring 2015	18.18	0.165	2.325	0.083	27	25.85	0.70	12.50	88.5	18	49	348
Sampling cycle 3												
Summer 2015	32.35	0.340	2.396	0.000	9	40.70	18.20	29.00	83	14	49	94
Autumn 2015	71.05	0.190	2.414	0.097	18	22.90	-2.20	11.40	89	21	55	136
Spring 2016	45.45	0.165	2.461	0.019	19	31.45	14.51	23.00	89	19	48	289


*Prevalence: Percentage of infected plants of mountain rue (*Ruta montana*).*

*S: Species richness*

*T_*max*_, T_*min*_, T_*mean*_: Average of monthly maximum, minimum, and mean temperatures (°C) in a season.*

*RH_*max*_, RH_*min*_, RH_*mean*_: Average of monthly maximum, minimum, and mean relative humidity (%) in a season.*

*Rainfalls: Average of total rainfalls (mm) in a season.*

**FIGURE 4:**
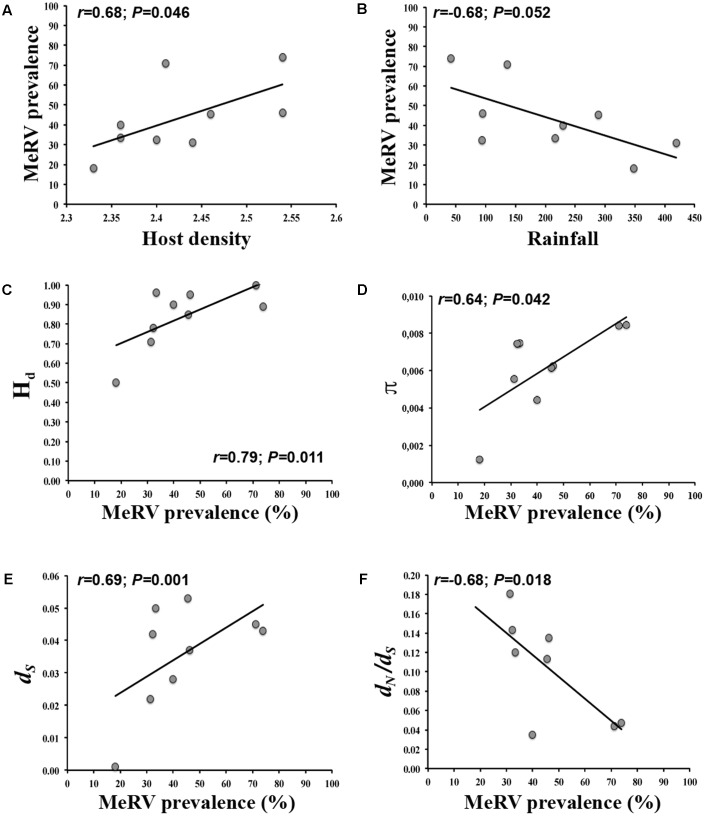
Bivariate relationships between MeRV prevalence/population evolutionary parameters and the best predictor variable. In each case, best predictor variable was identified by model selection analyses. Regressions of host density **(A)** and rainfall **(B)** on MeRV prevalence; and regressions of MeRV population haplotype diversity **(C)**, population genetic diversity **(D)**, number of synonymous mutations per site **(E)** and overall selection pressures *d*_N_*/d*_S_ **(F)** on MeRV prevalence (% of mountain rue infected plants) are represented. Note the different scales on the axes depending on the specific parameter analyzed.

To further understand the ecological determinants of MeRV infection risk in evergreen oak forest, we considered the following factors as predictors of MeRV prevalence: host plant density and relative abundance, species richness, plant biomass, temperature and relative humidity (minimal, maximal, and average values), and rainfall in the sampled locations (**Table 2**). We included season as a covariate to account for the dependency of MeRV prevalence over time. To analyze the association between these traits and MeRV prevalence, we used multiple regression model selection analyses (**Table 3**). The best-ranked model contained plant density as the only predictor, such that there was a positive association between mountain rue density and MeRV prevalence (*r* = 0.68; *P* = 0.046) (**Figure 4A**). This model closely competed with that containing rainfall as the only predictor, which showed a negative association between MeRV prevalence and rainfall (*r* = -0.68; *P* = 0.052) (**Table 3** and **Figure 4B**). The rest of the models showed much poorer predictive power (Δ*_i_* > 2) (see footnote of **Table 3**).

**Table 3:** Model selection analyses for MeRV prevalence, haplotype diversity (H_d_), genetic diversity (π), number of synonymous (*d*_S_) and non-synonymous (*d*_N_) substitutions per site, and selection pressures (*d*_N_*/d*_S_).

Model structure^∗^	*r*^†^	logLik	AIC^‡^	Δ*_i_*^§^	ω*_i_*^¶^
***Prevalence***					
Host plant density (100)	0.68^∗^	-22.44	52.87	0.00	0.52
Rainfall (100)	-0.68^∗^	-22.51	53.03	0.16	0.48
***H*_d_**					
MeRV prevalence (100)	0.79^∗^	7.60	-9.21	0.00	0.47
MeRV prevalence (90) + Plant species richness (10)	0.73^∗^	7.19	-8.38	0.83	0.30
MeRV prevalence (99) + Host relative abundance (1)	0.70^∗^	8.22	-7.67	1.51	0.22
**π**					
MeRV prevalence (77) + Plant species richness (23)	0.74^∗^	35.62	-63.23	0.00	0.47
MeRV prevalence (100)	0.64^∗^	36.35	-62.15	1.08	0.27
MeRV prevalence (99) + Rainfall (1)	0.43	37.00	-61.99	1.24	0.25
***d*_N_**					
MeRV prevalence (80) + Plant species richness (20)	0.70	45.44	-82.89	0.00	0.71
Plant species richness (100)	-0.65	45.54	-81.09	1.80	0.29
***d*_S_**					
MeRV prevalence (75) + Plant species richness (25)	0.82^∗^	29.60	-49.21	0.00	0.56
MeRV prevalence (100)	0.69^∗^	29.72	-47.43	1.78	0.23
MeRV prevalence (72) + Plant species richness (17) + Rainfall (11)	0.83	29.88	-47.30	1.91	0.21
***d*_N_*/d*_S_**					
MeRV prevalence (85) + Plant species richness (15)	0.83^∗^	30.34	-50.68	0.00	0.56
MeRV prevalence (78) + Plant species richness (13) + Rainfall (9)	0.84^∗^	30.44	-48.88	1.80	0.23
MeRV prevalence (100)	0.68^∗^	30.12	-48.75	1.93	0.21


*Model structures included host plant density, host relative abundance, plant species richness and plant biomass; and temperature, relative humidity, and rainfall in the sampled locations (minimal, maximal, and average values) as predictors, and season as covariate. MeRV prevalence was also included as predictor of virus evolution parameters. Best-ranked models are shown.*

*^∗^The relative importance (%) of each predictor variable is shown in parenthesis.*

*^†^Correlation coefficient. Asterisks indicate significant correlations (*P* < 0.05).*

*^‡^Akaike’s Information Criterion.*

*^§^Δ_*i*_, is the difference between the AIC of a given model and that of the best-ranked model, and quantifies how models compete (best-ranked model: Δ_*i*_ = 0; substantial empirical support: Δ_*i*_ = 1–2; considerable less support: Δ_*i*_ = 2–7; and no support; Δ_*i*_ > 10) (Burnham and Anderson, 2002).*

*^¶^AIC model weight as ω_*i*_ = exp(-0.5Δ_*i*_)/Σexp(-0.5Δ_*i*_). The larger the ω, the greater the likelihood of the model relatively to the competing models. Maximum ω = 1.*

### Analysis and Ecological Determinants of MeRV Population Genetic Diversity in Evergreen Oak Forests

Most of the MeRV sequenced isolates were collected at El Pardo, the location with the highest density of mountain rue plants. The low number of isolates from other locations prevented analyzing the genetic structure of the virus population according to geographic location. However, MCC trees indicated that isolates from location other than El Pardo did not form a monophyletic cluster (data available upon request). Hence, to test whether changes in ecological factors and MeRV prevalence across seasons affected the genetic diversity of the virus population, we only utilized the CP sequence of 69 MeRV isolates from El Pardo, collected along the three sampling cycles (**Table 4**). Using this sequence data set, we estimated the number of haplotypes (H), the haplotype diversity (H_d_) and the average nucleotide genetic diversity (π) of the MeRV population at each visit. MeRV population haplotype number, haplotype diversity and genetic diversity greatly varied between seasons (H = 2–9; H_d_ = 0.50–1.00, and π = 0.001–0.007) (**Table 4**).

**Table 4:** Number of haplotypes (H), haplotype diversity (H_d_), genetic diversity (π), and number of non-synonymous and synonymous substitutions (*d*_N_ and *d*_S_) per site of the El Pardo MeRV population based on the CP gene.

Season	N	H	H_d_ ± SE	π ± SE	*d_N_* ± SE	*d_S_* ± SE	*d_N_*/*d_S_* ± SE
Sampling cycle 1							
Summer 2013	7	6	0.952 ± 0.036	0.006 ± 0.002	0.005 ± 0.004	0.037 ± 0.012	0.135 ± 0.009
Autumn 2013	8	7	0.964 ± 0.027	0.007 ± 0.002	0.006 ± 0.004	0.050 ± 0.016	0.120 ± 0.012
Spring 2014	5	4	0.900 ± 0.072	0.004 ± 0.002	0.001 ± 0.000	0.028 ± 0.010	0.035 ± 0.000
*Cycle*^1^	20	16	0.974 ± 0.014	0.007 ± 0.002	0.015 ± 0.007	0.072 ± 8 × 10^-5^	0.097 ± 0.011
Sampling cycle 2							
Summer 2014	9	6	0.889 ± 0.030	0.008 ± 0.002	0.002 ± 0.002	0.043 ± 0.015	0.047 ± 0.002
Autumn 2014	7	4	0.714 ± 0.068	0.006 ± 0.002	0.004 ± 0.003	0.022 ± 0.009	0.181 ± 0.003
Spring 2015	4	2	0.500 ± 0.133	0.001 ± 0.001	0.003 ± 0.002	0.001 ± 0.000	3.000 ± 0.020
*Cycle*^1^	20	12	0.932 ± 0.020	0.005 ± 0.001	0.009 ± 0.020	0.066 ± 0.004	1.076 ± 0.020
Sampling cycle 3							
Summer 2015	4	4	1.000 ± 0.089	0.007 ± 0.002	0.006 ± 0.004	0.042 ± 0.014	0.143 ± 0.004
Autumn 2015	10	4	0.778 ± 0.029	0.008 ± 0.001	0.002 ± 0.002	0.045 ± 0.014	0.044 ± 0.002
Spring 2016	15	9	0.848 ± 0.023	0.006 ± 0.002	0.006 ± 0.004	0.053 ± 0.014	0.113 ± 0.004
*Cycle*^1^	29	16	0.931 ± 0.016	0.006 ± 0.001	0.018 ± 0.009	0.098 ± 0.024	0.100 ± 0.009


*^1^Data corresponding to group the sequences of the three stations of a sampling cycle.*

*N, number of sequences; H, number of haplotypes; H_*d*_, haplotype diversity; π, genetic diversity; SE, standard error.*

We analyzed the ecological factors affecting MeRV population H_d_, π, *d_N_*, *d_S_*, and *d_N_*/*d_S_* using again multiple regression model selection analyses. We utilized the same variables as for predicting prevalence but in this case, we added MeRV prevalence as an additional predictor (**Table 3**). The best-ranked model explaining H_d_ contained prevalence as the only predictor, and indicated a positive association between both traits (*r* = 0.79; *P* = 0.011) (**Table 3** and **Figure 4C**). This model closely competed with that including MeRV prevalence and plant species richness as main predictors (*r* = 0.73; *P* = 0.055), virus prevalence having much higher relative importance than plant species richness (89.8 and 10.2%, respectively). The model containing MeRV prevalence and host relative abundance also showed Δ*_i_* < 2 (*r* = 0.70; *P* = 0.046), with virus prevalence having again the highest relative importance (99.1%) (**Table 3**). In addition, the model best explaining MeRV population genetic diversity (π) contained MeRV prevalence and plant species richness as predictors (*r* = 0.74; *P* = 0.022), prevalence having the highest relative importance (77.2%). This model closely competed with that containing MeRV prevalence as the sole predictor (*r* = 0.64; *P* = 0.042) (**Figure 4D**), or this variable together with rainfall (*r* = 0.43; *P* = 0.541). However, this later model did not show a significant association between the predictors and the response variable (**Table 3**). None of the tested models accurately predicted *d_N_* (*P* ≥ 0.106) (**Table 3**). On the other hand, the best ranked models predicting *d_S_* and *d_N_*/*d_S_* contained MeRV prevalence as predictor, solely (**Figures 4E,F**) or in combination with plant species richness and rainfall. In all these models, MeRV prevalence had always much higher predictive power (relative importance ≥ 72%) than the other predictors (relative importance ≤ 25%) (**Table 3**).

## Discussion

Increasing evidence indicates that there is ample diversity of plant viruses in wild ecosystems. The analysis of the prevalence, genetic diversity and structure of plant virus populations has proven essential to understand the epidemiology and evolutionary biology of plant viruses, and to identify the determinants of virus populations dynamics and of the associated disease epidemics (reviewed by Burdon and Thrall, 2008; Pagán et al., 2016; Escriu, 2017). However, most of the virus species present in wild ecosystems remain uncharacterized (Roossinck, 2012; Stobbe and Roossinck, 2014), and the determinants of their epidemiology and evolution are largely unknown (Pagán et al., 2016). Here, we provide a detailed characterization of a new virus species isolated in evergreen oak forests of the Iberian Peninsula. This virus has the genome structure characteristic of the genus *Potyvirus*, for which the species demarcation criteria include: (1) different inclusion body morphology, (2) differences in host range, (3) overall nucleotide sequence identity not higher than 76%, and (4) capsid protein sequence identity not higher than 80% (Adams et al., 2005; King et al., 2012). Although we have not analyzed the inclusion body morphology, the reported characteristics of this virus fits with the other three demarcation criteria to be considered a new species in the genus *Potyvirus*, for which the name MeRV is proposed.

In evergreen oak forests, MeRV accounted for the largest proportion of potyvirus infections, and was highly prevalent in its only wild host: the perennial plant mountain rue (*Ruta montana*). Thus, in the analyzed ecosystem MeRV behaves as a specialist virus, i.e., viruses able to infect one or a few closely related host species (Futuyma and Moreno, 1988). This virus would therefore represent one of the first examples of its kind, as most plant viruses described in wild perennial plants naturally infect broader ranges of plants (Cooper and Jones, 2006). Specialist viruses are often extremely well adapted to their host(s). This means that these parasites are highly transmissible and efficiently exploit host resources to maximize its fitness (Woolhouse et al., 2001; García-Arenal and Fraile, 2013). Theoretical models of host adaptation assume that parasite transmission is positively correlated with its multiplication and negatively correlated with its virulence. In turn, multiplication is positively associated with virulence, which lead to a trade-off between transmission and virulence. Thus, these models predict that parasite fitness would be optimal at intermediate levels of virulence (Anderson and May, 1982). Despite behaving as specialist, MeRV infection did not induced any apparent symptom in mountain rue, neither in the field nor in the greenhouse. Similarly, infections by any of the other potyviruses detected in evergreen oak forests, which also showed narrow host ranges, were also asymptomatic. This would be in agreement with previous studies that generally reported a lack of obvious symptoms associated with virus infections in wild plants (Cooper and Jones, 2006; Roossinck, 2010; Prendeville et al., 2012). The apparent absence of symptoms in MeRV-infected mountain rue plants could be explained if: (i) MeRV is not optimally adapted to mountain rue, which is unlikely given the high prevalence of the virus; (ii) adaptation leads to reduced virulence; and (iii) negative effects of virus infection are not associated with plant growth but with other host traits. Some of our results support the later possibility. Given that infected plants cannot clear infection, and that mountain rue is a perennial plant, we would expect relatively homogeneous virus prevalence across seasons if virus adaptation would lead to reduced virulence (Fraile et al., 2017). However, MeRV prevalence was highly variable even within the same sampling cycle. This suggests that MeRV infection could have an effect on plant survival. Optimal temperature for seed germination of species from the *Ruta* genus is about 30°C (Mguis et al., 2011), and consequently young plants are more abundant in summer. Given that maximum MeRV prevalence occurs in this season and decays afterward, it could be hypothesized that MeRV-induced mortality could be more frequent in young individuals. In support of this hypothesis, additional surveys of mountain rue plants in evergreen oak forests indicated that MeRV was less prevalent in older plants (i.e., more than a year-old and at least one flowering period) (40%, 6/15 plants) than in younger plants (i.e., less than a year-old and not flowered) (71%, 5/7 plants). This indicates that not every young infected individual reaches the adult stage. Hence, our data strongly suggests that MeRV is an important modulator of the population dynamics of its host. On the other hand, this does not seem to be the case in crop hosts, as we failed in detecting the virus in pepper and tomato fields. Therefore, although a potential threat for these crops, MeRV infection in pepper and tomato is rare in the surveyed areas.

Focusing in the MeRV-mountain rue interaction, we analyzed the ecological factors affecting virus infection risk. Multivariate models indicated that host plant density was the major predictor of MeRV infection risk, such that the higher the host density the higher the virus prevalence. Therefore, our results support theory attributing a key role in infection risk to host density (Keesing et al., 2006; Ostfeld and Keesing, 2012), and are also in agreement with previous work in other plant virus–wild host interactions that also identified host density as a key factor for virus infection risk (Malmstrom et al., 2005; Borer et al., 2010; Pagán et al., 2012; Rodelo-Urrego et al., 2013). These theoretical and experimental works also identified ecosystem species diversity as a key determinant of virus infection risk. At odds, species diversity was not a good predictor of MeRV infection risk in wild mountain rue populations of evergreen oak forests. However, it should be noted that the effect of species diversity on infection risk is linked to its association with host density/abundance (Johnson et al., 2015). Interestingly, we did not find a significant association between density/abundance of mountain rue and number of plant species in evergreen oak forests (*r* ≤ 0.15; *P* ≥ 0.699), which could explain the poor predictive power of species diversity. Also, previous analyses identifying species diversity as a determinant of plant virus infection risk focused in viruses with a generalist strategy or at least with several hosts in the monitored ecosystem. The absence of alternative host species for MeRV in evergreen oak forests could minimize the effect of species diversity. These observations would support the idea that the applicability of the Dilution and the Amplification Effect hypotheses would depend on the plant community composition (Johnson et al., 2015). Finally, rainfall was negatively associated with MeRV prevalence. Higher MeRV prevalence was generally observed in summer, when rainfall is lower (see **Table 2**) and density of aphids (the main vectors of potyviruses) peaks (Nebreda et al., 2004; Mondal et al., 2016). Although we did not characterize MeRV transmission and the specific MeRV aphid vector species involved, our results would be compatible with the maximum activity of the associated MeRV vectors. This highlights the relevance of considering factors associated with virus transmission to fully understand the determinants of infection risk.

Epidemiological changes may result in genetic modifications in the parasite population (Grenfell et al., 2004; Archie et al., 2009; Pybus and Rambaut, 2009). Changes in MeRV prevalence were accompanied by variations in the virus population genetic and haplotype diversities. Viral populations may modify their genetic diversity by changing fixation rates, population sizes, and/or selection pressures (Moya et al., 2000). We could not analyze MeRV fixation rates, as the monitored time span was not sufficiently large for a meaningful estimate. However, given that the observed fluctuations in MeRV population genetic diversity occurred in short periods of time, it is unlikely that these are associated with changes in fixation rates. In turn, multiple regression model analyses indicated that MeRV prevalence was the best predictor of population genetic/haplotype diversity (higher virus genetic/haplotype diversities were observed at increasing prevalence). Higher virus prevalence results in increasing parasite population sizes (Burdon and Thrall, 2008). Therefore, our results suggest that MeRV genetic diversity is likely modulated by fluctuations in virus population size. This would be in agreement with theoretical elaborations predicting that higher population sizes may lead to higher genetic diversity (Scholle et al., 2013; Lanfear et al., 2014). Interestingly, MeRV prevalence was the best predictor of d_S_, both variables showing a positive association, but not of *d_N_*. Accordingly, selection pressures, measured as *d_N_*/*d_S_*, were negatively associated with virus prevalence. This indicates that neutral evolution, rather than adaptive selection, might be responsible for the changes in MeRV population genetic diversity associated with fluctuations in virus prevalence. Similar results have been obtained in other plant virus populations infecting wild hosts (Lima et al., 2013; Rodelo-Urrego et al., 2015).

Some cautionary comments, however, are called upon our results. First, although we conclude that MeRV is a specialist virus, we cannot discard that this virus is able to infect host species that, due to their low frequency in evergreen oak forests, have been insufficiently sampled. We believe, however, that this is unlikely given the sampling effort done in this work (over 10,000 samples). Second, the analyses of the association between MeRV prevalence and ecological factors are based on the data from six mountain rue populations, and the analyses of the association between MeRV evolutionary parameters and ecological/epidemiological factors are based on data from a single host population. We are aware that this might be a small sample size. However, it was enough to detect significant, and in many cases strong, correlations between the studied parameters. Third, the analysis of the effect of MeRV infection on host mortality is based on the differential virus prevalence between adult and young plants. It should be noted that these results could be also explained if virus titer in adult plants would be much lower than in younger ones, such that it could lead to an underestimation of virus prevalence in adults. However, no significant differences in MeRV accumulation were observed between young and adult plants (*F*_1,8_ = 0.63; *P* = 0.450). Fourth, although the best-ranked models explained a large proportion of the variation in MeRV infection risk and population genetic diversity, other factors not considered here could also play a role in MeRV evolution and epidemiology. For instance, host genetic diversity has been also reported as an important determinant of virus disease risk and population genetic diversity in wild ecosystems (Pagán et al., 2012; Rodelo-Urrego et al., 2015). Unfortunately, the lack of information on the mountain rue genomic sequence prevented including this variable in our analyses. Analyses in other host-pathogen system would help to tests the generality of our observations.

In summary, our results provide evidence of the relevant role that infections of native plant viruses may play in modulating the population dynamics of their hosts in wild ecosystems. The effect of the most important ecological factors driving MeRV infection risk and population genetic diversity in its wild host partially supports theoretical predictions. Deviations from these predictions could be at least partially attributed to the behavior of MeRV as specialist parasite. Because both infection risk and population genetic diversification have been proposed to be involved in the appearance of new infectious diseases (Keesing et al., 2006; Holmes, 2009), our results may contribute to understand the factors driving emergence.

## Author Contributions

CR-N collected and analyzed plant samples, obtained data on ecological factors and virus sequences, assembled the MeRV genome and wrote the manuscript. NM collected samples, performed multiple regression analyses, and participated in drafting the manuscript. IP designed and coordinated the study, helped collecting samples, and wrote the manuscript.

## Conflict of Interest Statement

The authors declare that the research was conducted in the absence of any commercial or financial relationships that could be construed as a potential conflict of interest.

## Footnotes

1 http://blast.ncbi.nlm.nih.gov/Blast.cgi

2 https://github.com/lh3/seqtk/

3 https://www.ncbi.nlm.nih.gov/orffinder/

4 https://www.bioinformatics.org/sms/prot_mw.html

5 http://darwin.uvigo.es/rdp/rdp.html
