# Supplementary material for: Ecological Factors Affecting Infection Risk and Population Genetic Diversity of a Novel Potyvirus in Its Native Wild Ecosystem
Source: Front Plant Sci. 2017 Nov 14;8:1958. doi: 10.3389/fpls.2017.01958 (PMC5694492; doi:10.3389/fpls.2017.01958)
Supplement: Supplementary file 1 [file Table_1.DOCX]

Supplementary Material

**Ecological factors affecting the infection risk and population genetic diversity of a novel potyvirus in its native wild ecosystem**

**Cristina Rodríguez-Nevado, Nuria Montes & Israel Pagán^*^**

*** Correspondence:** Dr. Israel Pagán: jesusisrael.pagan@upm.es

**Supplementary Table S1.** Locations in the Iberian Peninsula of evergreen oak forests and crop fields visited between 2013 and 2016.

| **Location** | **Abbreviation** | **Ecosystem** | **Latitude** | **Longitude** | **Elevation** |
| --- | --- | --- | --- | --- | --- |
| Carbonero el Mayor | Cem | Evergreen Oak | 41.148 | -4.318 | 928 |
| El Pardo | Par | Evergreen Oak | 40.501 | -3.740 | 616 |
| Montegancedo | Mon | Evergreen Oak | 40.403 | -3.836 | 713 |
| Cenicientos | Cen | Evergreen Oak | 40.259 | -4.449 | 773 |
| Aranjuez | Ara | Evergreen Oak | 40.020 | -3.599 | 563 |
| Marjaliza | Mar | Evergreen Oak | 39.578 | -3.922 | 853 |
|  |  |  |  |  |  |
| Cortijo de San Isidro | Csi-M | Cultivated/Melon | 40.054 | -3.580 | 494 |
| Villamanrique de Tajo | Vdt-M | Cultivated/Melon | 40.083 | -3.216 | 559 |
|  |  |  |  |  |  |
| Cortijo de San Isidro | Csi-T | Cultivated/Tomato | 40.056 | -3.579 | 496 |
| Ciruelos de Pradales | Cdp-T | Cultivated/Tomato | 41.439 | -3.705 | 1161 |
|  |  |  |  |  |  |
| Cortijo de San Isidro | Csi-P | Cultivated/Pepper | 40.055 | -3.579 | 495 |
| Ciruelos de Pradales | Cdp-P | Cultivated/Pepper | 41.439 | -3.703 | 1164 |
