# Supplementary material for: Ecological Factors Affecting Infection Risk and Population Genetic Diversity of a Novel Potyvirus in Its Native Wild Ecosystem
Source: Front Plant Sci. 2017 Nov 14;8:1958. doi: 10.3389/fpls.2017.01958 (PMC5694492; doi:10.3389/fpls.2017.01958)
Supplement: Supplementary file 2 [file Table_2.DOCX]

Supplementary Material

**Ecological factors affecting the infection risk and population genetic diversity of a novel potyvirus in its native wild ecosystem**

**Cristina Rodríguez-Nevado, Nuria Montes & Israel Pagán^*^**

*** Correspondence:** Dr. Israel Pagán: jesusisrael.pagan@upm.es

**Supplementary Table S2.** Specific primers designed to obtain the full-length genomic sequence of MeRV-ParP17.

| **Primer** | **Sequence (5'–3')** | **Position^1^** |
| --- | --- | --- |
| MERV-P1 5UTR For | CGTATCATCAGAGACCCGTG | 100-119 |
| MERV-P1 5UTR Rev | CACGTACGTGTTGGAGAGCC | 314-333 |
| MERV-P1For | CATGTGAAACTGATCTTAA | 223-241 |
| MERV-P1Rev | CTATTGCAAGGGAATAGTGC | 1185-1204 |
| MERV-For1 | GTAGCTAGCTGTGGTTCGG | 997-1015 |
| MERV-Rev1 | CCTTTGAGCGTTCCTCCGAC | 1968-1987 |
| MERV-For2 | GCAACTTCGAAGCAGCAAAG | 1853-1872 |
| MERV-Rev2 | CTGCACACTCCATGACCG | 2812-2829 |
| MERV-For3 | GCGAGTTAGATCAGTGTTG | 2753-2771 |
| MERV-Rev3 | CACCGCGGACTAGTATGTC | 3709-3727 |
| MERV-For4 | CTAAGAGGGAGTCAGACAAC | 3553-3572 |
| MERV-Rev4 | CTACACGTCCAAGTCTCTG | 4522-4540 |
| MERV-For5 | GAAGGTGTCGTTGATTTCG | 4420-4438 |
| MERV-Rev5 | GCATCGTGAGCTAACAATG | 4529-5447 |
| MERV-For6 | GTGCGAGCTCAGTTGATGG | 5278-5296 |
| MERV-Rev6 | GGACACTTGAAGATACCAT | 6287-6305 |
| MERV-For7 | CATGCGCGATTACAATCC | 6126-6143 |
| MERV-Rev7 | GTCCAACGATAATCGGGTTTG | 7067-7087 |
| MERV-For8 | GCTAAATTCTTCAGGCCTC | 6982-7000 |
| MERV-Rev8 | CTTGAGACCTAGTTCAGC | 7954-7971 |
| MERV-For9 | GTACTGAAGAGATTGACG | 7844-7861 |
| MERV-Rev9 | CCGTCCATCATTGTCCAG | 8826-8843 |
| MERV-For10 | GTATGATGGCGTGAAAGCG | 8712-8730 |
| MERV-Rev10 | GTAGCACCTCACTAACAAG | 9288-9306 |

^1^ Positions according to the NGS-derived MeRV nucleotide genomic sequence.
