# Supplementary material for: Ecological Factors Affecting Infection Risk and Population Genetic Diversity of a Novel Potyvirus in Its Native Wild Ecosystem
Source: Front Plant Sci. 2017 Nov 14;8:1958. doi: 10.3389/fpls.2017.01958 (PMC5694492; doi:10.3389/fpls.2017.01958)
Supplement: Supplementary file 3 [file Table_3.DOCX]

Supplementary Material

**Ecological factors affecting the infection risk and population genetic diversity of a novel potyvirus in its native wild ecosystem**

**Cristina Rodríguez-Nevado, Nuria Montes & Israel Pagán^*^**

*** Correspondence:** Dr. Israel Pagán: jesusisrael.pagan@upm.es

**Supplementary** **Table S3.** Species of the genus *Potyvirus* compiled from GenBank used in the phylogenetic analyses.

| **GenBank Acc. number** | **Virus name** |
| --- | --- |
| NC_010736 | *Algerian watermelon mosaic virus* |
| NC_014905 | *Apium virus Y* |
| NC_018176 | *Arracacha mottle virus* |
| NC_025821 | *Asparagus virus 1* |
| NC_009745 | *Banana bract mosaic virus* |
| NC_009741 | *Basella rugose mosaic virus* |
| NC_004047 | *Bean common mosaic necrosis virus* |
| NC_003397 | *Bean common mosaic virus* |
| NC_003492 | *Bean yellow mosaic virus* |
| NC_005304 | *Beet mosaic virus* |
| NC_023014 | *Bidens mosaic virus* |
| NC_014325 | *Bidens mottle virus* |
| NC_019415 | *Blue squill virus A* |
| NC_030847 | *Brazilian weed virus Y* |
| NC_014536 | *Brugmansia suaveolens mottle virus* |
| NC_021196 | *Calla lily latent virus* |
| NC_030794 | *Callistephus mottle virus* |
| NC_013261 | *Canna Yellow Streak Virus* |
| NC_025254 | *Carrot thin leaf virus* |
| NC_027210 | *Catharanthus mosaic virus* |
| NC_015393 | *Celery mosaic virus* |
| NC_016044 | *Chilli ringspot virus* |
| NC_005778 | *Chilli veinal mottle virus* |
| NC_003536 | *Clover yellow vein virus* |
| NC_003742 | *Cocksfoot streak virus* |
| NC_020072 | *Colombian datura virus* |
| NC_004013 | *Cowpea aphid-borne mosaic virus* |
| NC_008028 | *Daphne mosaic virus* |
| NC_003537 | *Dasheen mosaic virus* |
| NC_021197 | *Donkey orchid virus A* |
| NC_007728 | *East Asian Passiflora virus* |
| NC_034273 | *Endive necrotic mosaic virus* |
| NC_031339 | *Euphorbia ringspot virus* |
| NC_014064 | *Freesia mosaic virus* |
| NC_010954 | *Fritillary virus Y* |
| NC_021786 | *Habenaria mosaic virus* |
| NC_015394 | *Hardenbergia mosaic virus* |
| NC_017967 | *Hippeastrum mosaic virus* |
| NC_030236 | *Impatiens flower break potyvirus* |
| NC_018833 | *Iranian johnsongrass mosaic virus* |
| NC_029076 | *Iris severe mosaic virus* |
| NC_000947 | *Japanese yam mosaic virus* |
| NC_029051 | *Jasmine ringspot virus* |
| NC_003606 | *Johnsongrass mosaic virus* |
| NC_007913 | *Konjac mosaic virus* |
| NC_004011 | *Leek yellow stripe virus* |
| NC_003605 | *Lettuce mosaic virus* |
| NC_005288 | *Lily mottle virus* |
| NC_014898 | *Lupine mosaic virus* |
| NC_003377 | *Maize dwarf mosaic virus* |
| NC_009995 | *Moroccan watermelon mosaic virus* |
| NC_008824 | *Narcissus degeneration virus* |
| NC_023628 | *Narcissus late season yellows virus* |
| NC_011541 | *Narcissus yellow stripe virus* |
| NC_005029 | *Onion yellow dwarf virus* |
| NC_019409 | *Ornithogalum mosaic virus* |
| NC_014252 | *Panax virus Y* |
| NC_005028 | *Papaya leaf-distortion mosaic potyvirus* |
| NC_001785 | *Papaya ringspot virus* |
| NC_014790 | *Passion fruit woodiness virus* |
| NC_001671 | *Pea seed-borne mosaic virus* |
| NC_002600 | *Peanut mottle virus* |
| NC_007147 | *Pennisetum mosaic virus* |
| NC_001517 | *Pepper mottle virus* |
| NC_008393 | *Pepper severe mosaic virus* |
| NC_011918 | *Pepper veinal mottle virus* |
| NC_014327 | *Pepper yellow mosaic virus* |
| NC_004573 | *Peru tomato mosaic virus* |
| NC_001445 | *Plum pox virus* |
| NC_018872 | *Pokeweed mosaic virus* |
| NC_004039 | *Potato virus A* |
| NC_004010 | *Potato virus V* |
| NC_001616 | *Potato virus Y* |
| NC_003399 | *Scallion mosaic virus* |
| NC_007433 | *Shallot yellow stripe virus* |
| NC_004035 | *Sorghum mosaic virus* |
| NC_002634 | *Soybean mosaic virus* |
| NC_003398 | *Sugarcane mosaic virus* |
| NC_014038 | *Sunflower chlorotic mottle virus* |
| NC_034208 | *Sunflower ring blotch virus* |
| NC_001841 | *Sweet potato feathery mottle virus* |
| NC_020896 | *Sweet potato latent virus* |
| NC_017970 | *Sweet potato virus 2* |
| NC_014742 | *Sweet potato virus C* |
| NC_018093 | *Sweet potato virus G* |
| NC_026615 | *Tamarillo leaf malformation virus* |
| NC_009742 | *Telosma mosaic virus* |
| NC_007180 | *Thunberg fritillary virus* |
| NC_001555 | *Tobacco etch virus* |
| NC_009994 | *Tobacco vein banding mosaic virus* |
| NC_001768 | *Tobacco vein mottling virus* |
| NC_017824 | *Tomato necrotic stunt virus* |
| NC_002509 | *Turnip mosaic virus* |
| NC_017977 | *Vallota speciosa virus* |
| NC_025250 | *Vanilla distortion mosaic virus* |
| NC_010735 | *Verbena virus Y* |
| NC_006262 | *Watermelon mosaic virus* |
| NC_004426 | *Wild potato mosaic virus* |
| NC_009744 | *Wild tomato mosaic virus* |
| NC_007216 | *Wisteria vein mosaic virus* |
| NC_016441 | *Yam bean mosaic virus* |
| NC_019412 | *Yam mild mosaic virus* |
| NC_004752 | *Yam mosaic virus* |
| NC_011560 | *Zantedeschia mild mosaic virus* |
| NC_023175 | *Zucchini tigre mosaic virus* |
| NC_003224 | *Zucchini yellow mosaic virus* |
